# Massage Alleviates Delayed Onset Muscle Soreness after Strenuous Exercise: A Systematic Review and Meta-Analysis

**DOI:** 10.3389/fphys.2017.00747

**Published:** 2017-09-27

**Authors:** Jianmin Guo, Linjin Li, Yuxiang Gong, Rong Zhu, Jiake Xu, Jun Zou, Xi Chen

**Affiliations:** ^1^School of Kinesiology, Shanghai University of Sport, Shanghai, China; ^2^School of Sports Science, Wenzhou Medical University, Wenzhou, China; ^3^Wenzhou People's Hospital, The Third Clinical Institute of Wenzhou Medical University, Wenzhou, China; ^4^Molecular Laboratory, School of Pathology and Laboratory Medicine, The University of Western Australia, Perth, Australia

**Keywords:** massage, physiotherapy, exercise, delayed onset muscle soreness (DOMS), systematic review, meta-analysis, PROSPERO registration number: CRD42016053118.

## Abstract

**Purpose:** The purpose of this systematic review and meta-analysis was to evaluate the effects of massage on alleviating delayed onset of muscle soreness (DOMS) and muscle performance after strenuous exercise.

**Method:** Seven databases consisting of PubMed, Embase, EBSCO, Cochrane Library, Web of Science, CNKI and Wanfang were searched up to December 2016. Randomized controlled trials (RCTs) were eligible and the outcomes of muscle soreness, performance (including muscle maximal isometric force (MIF) and peak torque) and creatine kinase (CK) were used to assess the effectiveness of massage intervention on DOMS.

**Results:** Eleven articles with a total of 23 data points (involving 504 participants) satisfied the inclusion criteria and were pooled in the meta-analysis. The findings demonstrated that muscle soreness rating decreased significantly when the participants received massage intervention compared with no intervention at 24 h (SMD: –0.61, 95% CI: –1.17 to –0.05, *P* = 0.03), 48 h (SMD: –1.51, 95% CI: –2.24 to –0.77, *P* < 0.001), 72 h (SMD: –1.46, 95% CI: –2.59 to –0.33, *P* = 0.01) and in total (SMD: –1.16, 95% CI: –1.60 to –0.72, *P* < 0.001) after intense exercise. Additionally, massage therapy improved MIF (SMD: 0.56, 95% CI: 0.21–0.90, *P* = 0.002) and peak torque (SMD: 0.38, 95% CI: 0.04–0.71, *P* = 0.03) as total effects. Furthermore, the serum CK level was reduced when participants received massage intervention (SMD: –0.64, 95% CI: –1.04 to –0.25, *P* = 0.001).

**Conclusion:** The current evidence suggests that massage therapy after strenuous exercise could be effective for alleviating DOMS and improving muscle performance.

## Introduction

It is well established that keeping an active lifestyle through exercise benefits human health, especially reducing the risk of obesity and cardiovascular disease. However, exhaustive or unaccustomed exercise (particularly involving eccentric contractions) frequently result in temporary muscle damage, leading to delayed onset muscle soreness (DOMS) (Bleakley et al., 2012).

DOMS commonly occurs within the first 24 h after exhaustive or intense exercise, reaching a peak between 24 and 72 h (Howatson and van Someren, 2008). It is often accompanied by muscle swelling and reduction in muscle performance (Kargarfard et al., 2016; De Marchi et al., 2017), as well as a decrease in range of motion (Cheung et al., 2003; Lavender and Nosaka, 2006). Although the exact mechanism of DOMS remains unclear, the most accepted theory suggests primary mechanical damage induced by exercise, followed by inflammation attributing to the symptoms of DOMS. This is verified by microscopic analysis showing disruption of muscle fibers. In addition, there is also an increase of intracellular enzymes such as creatine kinase (CK) and inflammatory markers in blood (Peake et al., 2005a,b; Chatzinikolaou et al., 2010).

In an attempt to prevent or alleviate the symptoms of DOMS, a number of conventional physiotherapeutic strategies were used such as massage, cold-water immersion, whole body vibration, compression garments, and stretching (Weerapong et al., 2005; Howatson et al., 2009; Bleakley et al., 2012; Costello et al., 2012; Hill et al., 2014). Therapeutic massage has been used for body health for thousands of years worldwide. As a physiotherapeutic intervention, massage treatment is widely used to alleviate clinical symptoms of DOMS, and to benefit the athlete's recovery after exercise in preparation for the next event (Poppendieck et al., 2016). The potential effectiveness of massage therapy is proposed to increase skin and muscle temperature, blood and lymphatic flow, and parasympathetic activity. Subsequently, the effects include then relief of muscle tension and stiffness, reduction of muscle soreness, and increased joint range of motion (Weerapong et al., 2005). In addition, changes in the Hoffman reflex after massage also contributes to the reduction of muscle pain (Morelli et al., 1999). Moreover, the psychophysiological response to massage, including relaxation, leads to mood enhancement and fatigue reduction (Hemmings et al., 2000).

Previous systematic reviews have shown benefits from massage for symptoms of DOMS after intense exercise. However, little support for the use of massage was observed to enhance muscle performance (Ernst, 1998; Best et al., 2008), including peak torque and maximal isometric force, factors that are important for sporting events. It seems that the psychological effect is larger than the physiological effect. Torres et al. demonstrated that massage could reduce muscle soreness and increase muscle recovery 24 h post-exercise based on a meta-analysis, but there were only few trials pooled in the meta-analysis (3 trials) (Torres et al., 2012). Currently, the evidence of the effects of massage intervention on DOMS still limited. Therefore, the purpose of this study was to evaluate the effectiveness of massage on DOMS and muscle performance after strenuous exercise.

## Method

### Search strategy

Relevant research articles from January 1980 to December 2016 were collected with keywords such as “exercise,” “massage,” “delayed onset muscle soreness,” and “random” from the following databases: PubMed, Embase, EBSCO, Cochrane Library, Web of Science, China Knowledge Resource Integrated Database (CNKI) and Wanfang Database. The search was imposed with the limitation of randomized controlled trials (RCTs), and without language or status limitations. Supplementary Data (S1) details all of the search strategies used in this study. The protocol was registered on the international prospective register of systematic reviews PROSPERO (http://www.crd.york.ac.uk/PROSPERO), registration number: CRD42016053118.

### Inclusion criteria

Trials were included when they met the inclusion criteria as follows: (a) only the trials designed as RCTs were covered, (b) participants were human with no muscle or bone diseases, (3) the trials compared post-exercise massage intervention with the control group receiving usual care, or no intervention, (4) outcomes included the primary outcome of muscle pain or soreness rating, the secondary outcome: muscle strength (i.e., maximal isometric force (MIF) and peak torque), and the level of serum CK. Mean and standard deviation (SD) were reported in the trials.

### Exclusion criteria

Trials were excluded when they met the exclusion criteria as the following: (a) the articles were conference posters, abstracts, or case reports; (b) mean and SD could not be obtained from the articles or authors.

### Studies selection

Two reviewers (Guo JM and Li LJ) independently reviewed the titles or abstracts of all studies, and the full contents of the relevant studies were checked carefully to evaluate whether the study could be included. Any disagreements were resolved by discussion or consultation with a third author (Chen X) if necessary.

### Quality assessment

The Cochrane Collaboration tool was used to evaluate the risk of bias of the included trials (Higgins et al., 2011). Two reviewers (Guo JM and Li LJ) independently evaluated seven domain biases as follows: random sequence generation (selection bias), allocation concealment (selection bias), blinding of participants and personnel (performance bias), blinding of outcome assessment (detection bias), incomplete outcome data (attrition bias), and selective reporting (reporting bias). Three grades of high, low, or unclear bias were labeled for every study included. Disagreements were resolved by discussion or consulting with a third independent reviewer (Chen X) if necessary.

### Data extraction

The two reviewers independently extracted the data from every included eligible trial as the following: study characteristics (i.e., author and year), participant characteristics (i.e., age and number of participants), description of interventions, study period, outcomes, and time points. Any disagreements were settled by discussion to reach unanimity, and the authors of the trials were contacted directly to acquire original studies and data if necessary.

### Statistical analysis

The Review Manager software (RevMan 5.3; Cochrane Collaboration) was used to perform the meta-analysis. Heterogeneity among studies was evaluated by I^2^ statistic as follows: low heterogeneity was assumed when I^2^ < 25%; moderate heterogeneity when I^2^ < 75% and >25%; high heterogeneity when I^2^ ≥ 75% (Higgins et al., 2003). Meta-analysis was used to combine two or more outcomes from the studies by random-effects or by a fixed-effect model. The fixed-effect model was used when I^2^ < 25% and the random-effects model was used if I^2^ > 25%. As the trials used different scales for muscle soreness rating assessment, we used standardized mean difference (SMD) to analyze the effects. If *P* < 0.05, it was considered to be a significant difference. Subgroups were used to analyze the effectiveness of massage at different time points after strenuous exercise. The possible publication bias was evaluated by Funnel plot asymmetry if more than nine trials were included. Sensitivity analysis was used when I^2^ > 50%, removing each trial one by one to evaluate the stability of the results.

If the included articles did not report mean and SD, we would contact the authors to ask for data, and the articles would be excluded if there was no reply. When the articles showed the data as median and interquartile range (IQR), the mean would be equivalent to the median, and the SD would be calculated as *SD* = IQR/1.35 (Wang et al., 2016). If the articles reported the data in terms of mean and standard error (SE), the SD would be calculated as SD = SE$\times \sqrt{n}\;$(n meaning sample size) (Higgins and Green, 2011; Higgins et al., 2011).

## Results

### Search results

**Figure 1:**
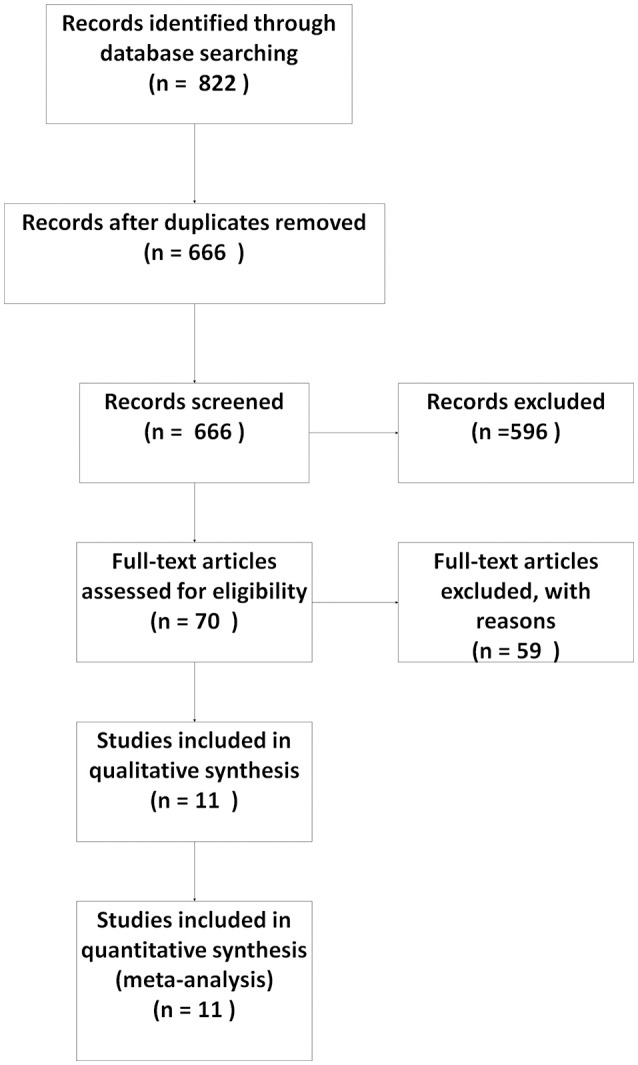
Flow diagram of the study selection.

From our initial search, 882 records were obtained. After reviewing the information on the articles' title and abstract, 70 potentially eligible articles were identified. After reviewing the full content of the studies, 11 articles with 23 data points (504 participants) satisfied the inclusion criteria and were pooled in this meta-analysis (Weber et al., 1994; Lightfoot et al., 1997; Hilbert et al., 2003; Mancinelli et al., 2006; Yang, 2007; Frey Law et al., 2008; Xiong et al., 2009; Hu et al., 2010; Andersen et al., 2013; Imtiyaz et al., 2014; Kargarfard et al., 2016). The details of the process of identifying articles from initially searching to inclusion are shown in Figure 1.

### Description of included studies

The characteristics of the included articles were summarized in Table 1. Eleven articles were included in this meta-analysis. The distribution of publication countries was as follows: the United States (*n* = 5, 45.5%), the People's Republic of China (*n* = 3, 27.2%), Canada (*n* = 1, 9.1%), India (*n* = 1, 9.1%) and Iran (*n* = 1, 9.1%). Eight articles were published in English, and three in Chinese.

**Table 1:** Characteristics of included studies.

**No**	**Article, year**	**Country/Region**	**Subjects' characteristic, sample size**	**Induce DOMS**	**Intervention**	**Duration**	**Outcomes**	**Time point**
1	Weber et al., 1994	USA	20 healthy female untrained volunteers (G1 = 10, G2 = 10). Mean age(SD): G1 = 22.3(4) years, G2 = 25.9(4.5) years	Performed eccentric exercise of nondominant elbow flexion until fatigued	G1: no intervention, G2:massage, light effleurage for 2 min, petrissage for 5 min, effleurage for 1 min.	8 min	Muscle soreness rating, maximal isometric force, peak torque at 60°/s	0, 24, 48 h after DOMS induced exercise
2	Lightfoot et al., 1997	USA	21 normally active college-age volunteers [G1 = 11 (3 men and 8 women), G2 = 10 (6 men and 4 women)]. Mean age(SD): G1 = 23.9(6.4) years, G2 = 26.9(5.6) years	4 sets of 15 repetitions of eccentric exercise (heel-drop) to induce calf muscle DOMS	G1: no intervention, G2: 10 min of petrissage on calf muscle immediately after eccentric exercise, and again at 24 h post-exercise.	10 min twice	Muscle soreness rating, creatine kinase level	0, 24, 48 h after DOMS induced exercise
3	Hilbert et al., 2003	USA	18 male and female volunteers (G1 = 9, G2 = 9).mean age (SD) for all the subjects is 20.4 (1.0) years	6 sets of 10 maximal eccentric contractions with hamstring (1 min of rest between sets), followed by 5 more maximal eccentric contractions.	G1:rest for 20 min while listening to the same audiotape heard by the massage group on placebo lotion. G2:20 min of massage classical Swedish techniques	20 min	Muscle soreness rating, peak eccentric torque	0, 2, 6, 24, 48 h after DOMS induced exercise
4	Mancinelli et al., 2006	USA	22 female basketball and volleyball players. G1 = 11, G2 = 11. Mean age (SD) for all subjects is 20(0.93) years	Intense strength training and drills	G1: no intervention. G2: Western massage techniques of effleurage, petrissage and vibration were used for 17 min on 48 h after DOMS induced training.	17 min	Muscle soreness rating (VAS and PPT)	0 h after treatment (48 h after DOMS induced training)
5	Yang, 2007	China	16 male trained athletic volunteers (G1 = 8, G2 = 8), mean age (SD) for all subjects is 19.2(0.96) years	10 sets of 30 m leapfrog with 1–2 min rest between sets.	G1: no intervention. G2: Chinese traditional massage (including pushing, grasping, vibrating, and plucking) for 15 min.	15 min	Muscle soreness rating, creatine kinase level	0, 24 h after DOMS induced exercise
6	Frey Law et al., 2008	USA	27 healthy individuals participanted in this study as volunteers, G1 = 11, G2 = 16.mean age (SD) for all subjects is 23.3 (3.5) years, range of 19–41 years	3 sets of eccentric wrist extensor contractions using a 10 lb hand weight with 1–2 min between sets.	G1: had a thin layer of massage cream applied but no massage received. G2: received a deep-tissue massage of the fore-arm (including effleurage for 2 min and petrissage for 4 min)	6 min	Muscle soreness rating (VAS and PPT), Peak Torque	24–48 h after DOMS induced exercise
7	Xiong et al., 2009	China	20 healthy male untrained students (G1 = 10, G2 = 10). Mean age (SD): G1 = 23.40(2.88) years, G2 = 23.20(2.90) years.	2 sets of eccentric nondominant elbow flexion with 60% maximal isometric force. 25 repetitions for each set with 5 min rest between sets.	G1: no intervention. G2: received Chinese traditional massage (including pushing, swing, grasping, vibrating and plucking) for 30 min each day with 3 days post-exercise.	30 min for every day	Muscle soreness rating, creatine kinase level	0, 24, 48, 72 h after DOMS induced exercise
8	Hu et al., 2010	China	15 healthy male students (G1 = 8, G2 = 7). Mean age (SD): G1 = 22.13(3.23) years, G2 = 22.14(3.08) years.	7 sets of 50 m leapfrog with 2–3 min rest between sets.	G1: no intervention. G2: Chinese traditional massage (including pushing, grasping, vibrating and plucking) for 20 min each day with 5 days post-exercise.	20 min for every day	Mucle soreness rating, creatine kinase level, maximal isometric force, Peak Torque at 120°/s	0, 2, 24, 48, 72, 96 h after DOMS induced exercise
9	Andersen et al., 2013	Canada	20 female volunteers, G1 = 10, G2 = 10. Mean age (SD) for all subjects is 32(11) years.	10 sets of 10–15 repetitions of maximal eccentric contractions of upper trapezius, with 1 min rest between sets.	G1: performed unilateral shoulder shrugs with the Thera-Band elastic tubing. G2: 10 min massage on the trapezius muscle (including petrissage and effleurage) on 48 h after DOMS induced training.	10 min	Mucle soreness rating (VAS and PPT)	0 h after treatment (48 h after DOMS induced training)
10	Imtiyaz et al., 2014	India	30 healthy female non athletic subjects, G1 = 15, G2 = 15. Mean age (SD): G1 = 19.66 (1.01) years, G2 = 20.33 (0.97) years.	30 repetitions of eccentric exercise of elbow flexion using a dumbbell with weight of 80% maximal isometric force.	G1: no intervention. G2: received therapeutic massage for 15 min	15 min	Mucle soreness rating, creatine kinase level, maximal isometric force	24, 48, 72 h after DOMS induced exercise
11	Kargarfard et al., 2016	Iran	30 healthy males with at least 2 years experience in bodybuilding, G1 = 15, G2 = 15. Mean age (SD): G1 = 28.07 (3.33) years, G2 = 29.47 (3.72) years.	Performed squats or leg press to 90° knee flexion for five sets at 75–77% 1RM until exhaustion, with 1 min rest between sets	G1: no intervention. G2: Western massage techniques of effleurage, petrissage and vibration were used for 30 min	30 min	Mucle soreness rating, creatine kinase level, maximal isometric force	0, 24, 48, 72 h after intervention

*VAS, Visual analog scale; PPT, pressure pain threshold*.

### Massage therapy

Most (eight articles) of the trials used western massage techniques or Swedish massage techniques including effleurage and petrissage. Effleurage is a succession of light and stroking massage, and petrissage is deep-tissue kneading of the muscles. Three articles performed traditional Chinese massage including pushing, swing, grasping, vibrating and plucking. According to the size of the muscle area, the massage intervention duration ranged from 6 to 30 min, but the duration per area approximately similar between the trials (i.e., 6 min massage for wrist muscles, 30 min massage for lower extremity muscles).

### Control conditions

Most (eight articles) of the studies used no intervention (just sitting for rest). The participants in one study listened to audiotapes while resting, while one study had a thin layer of massage cream applied but no massage received. Another study performed unilateral shoulder shrugs with Thera-Band elastic tubing.

### Risk of bias of included studies

**Figure 2:**
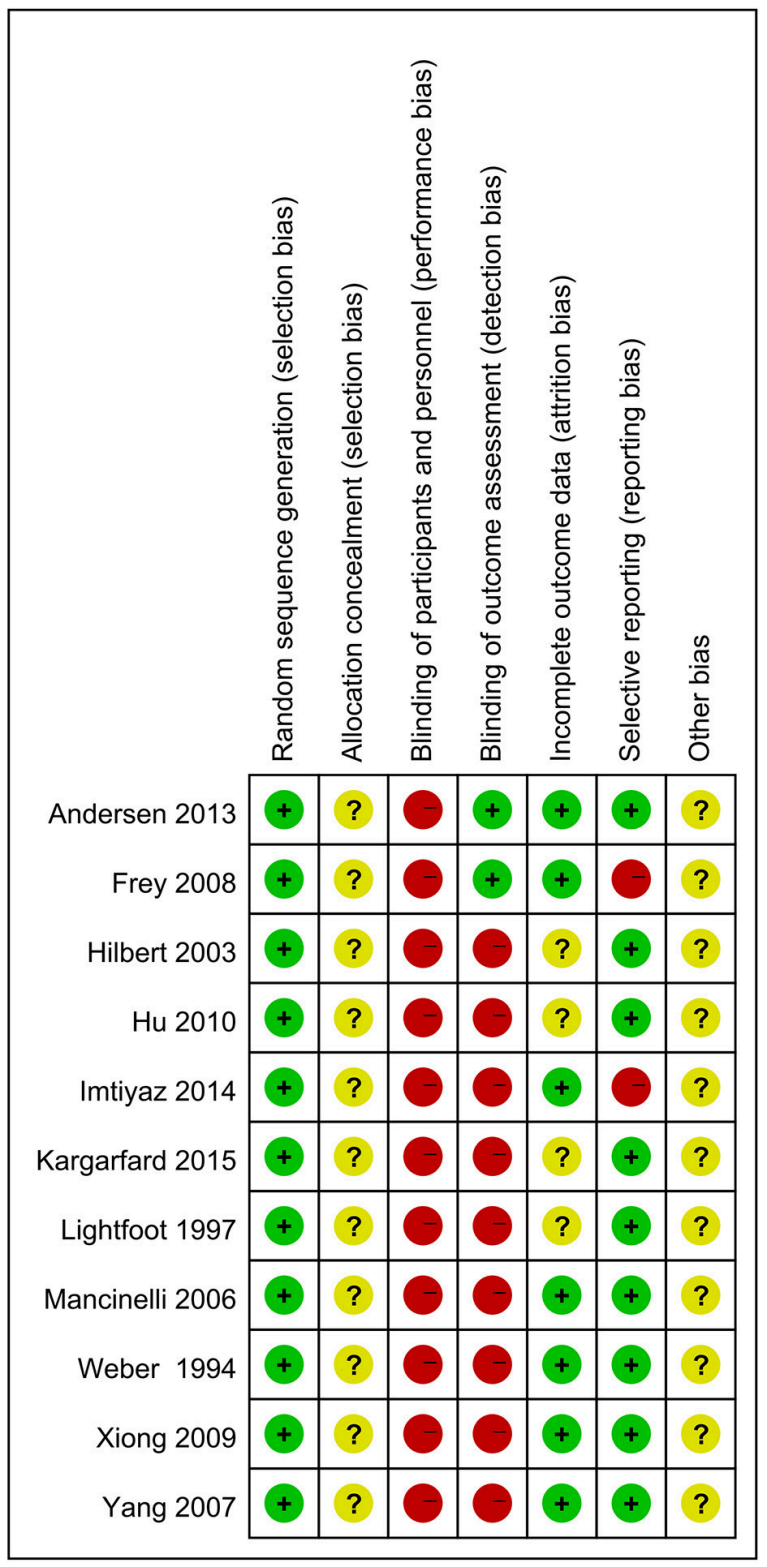
Risk of bias summary of included studies.

Every included study was assessed for the risk of bias according to instructions by Higgins and Green (2011); Higgins et al. (2011). As shown in Figure 2, all of the 11 articles used a randomization method, but none of these reported any information about allocation concealment. None of the trials met the requirements for the blinding of participants. However, it seems unfeasible to use the blinding method in view of the massage intervention. Only two studies (18.2%) masked their outcome assessors, which increased the risk of detection bias. Seven studies (63.6%) showed low risk of incomplete outcome bias, and the other four were unclear. Nine studies (81.8%) showed a low risk bias of selective reporting, whereas it was unclear if the studies have additional bias. Funnel plot asymmetry did not show any publication bias of muscle soreness rating, MIF, or the serum levels of CK (Supplemental Figures S1–S3).

### Effects of massage on muscle soreness rating

**Figure 3:**
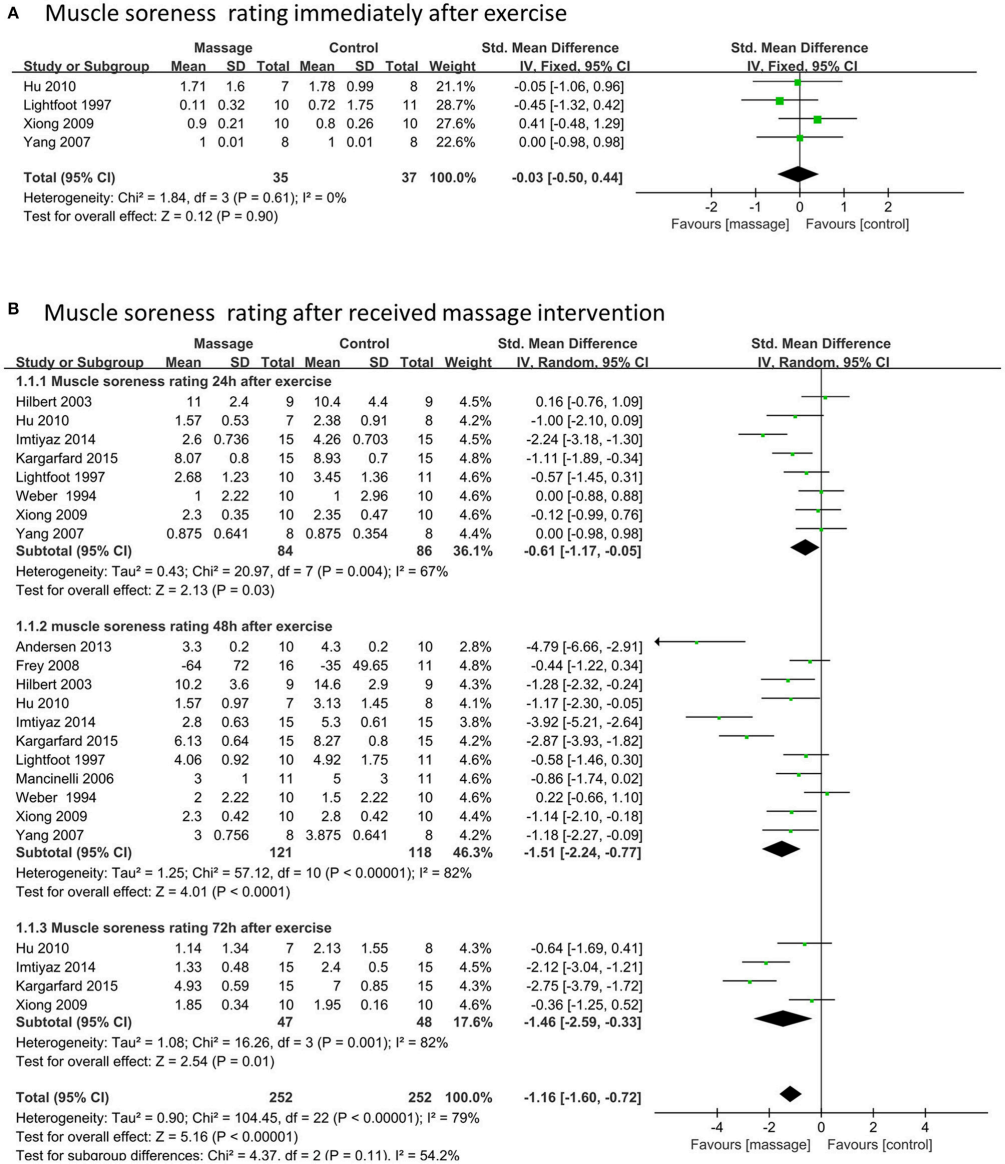
Meta-analysis of effects of massage intervention on muscle soreness rating. **(A)** The time point immediately. **(B)** Time points after received massage intervention combined 24, 48, and 72 h after exercise.

Four data points (72 participants) reported a muscle soreness rating immediately after strenuous exercise. No significant differences were found when the massage and control groups were compared based on a fixed model (SMD –0.03, 95%CI 0.5–0.44, *P* = 0.90, I^2^ = 0%) (Figure 3A). Twenty-three data points (504 participants) showed that a muscle soreness rating significantly decreased after the participants received massage intervention when compared with no intervention (SMD –1.16, 95%CI –0.72 to –1.60, *P* < 0.001, I^2^ = 79%) (Figure 3B). Among these, the muscle soreness rating presented a significant decrease compared to the control group when the participants received massage intervention in subgroups of 24 h (SMD –0.61, 95%CI –1.17 to –0.05, *P* = 0.03, I^2^ = 67%), 48 h (SMD –1.51, 95%CI –2.24 to –0.77, *P* < 0.0001, I^2^ = 82%) and 72 h (SMD –1.46, 95%CI –2.59 to –0.33, *P* = 0.01, I^2^ = 82%) post-exercise, respectively (Figure 3B). Based on these results, massage intervention after exercise showed higher efficacy at 48 and 72 h after exercise than at 24 h.

Sensitivity analysis revealed that outcomes of the total effect of muscle soreness rating were stable when trials were removed one by one.

### Effects of massage on MIF

**Figure 4:**
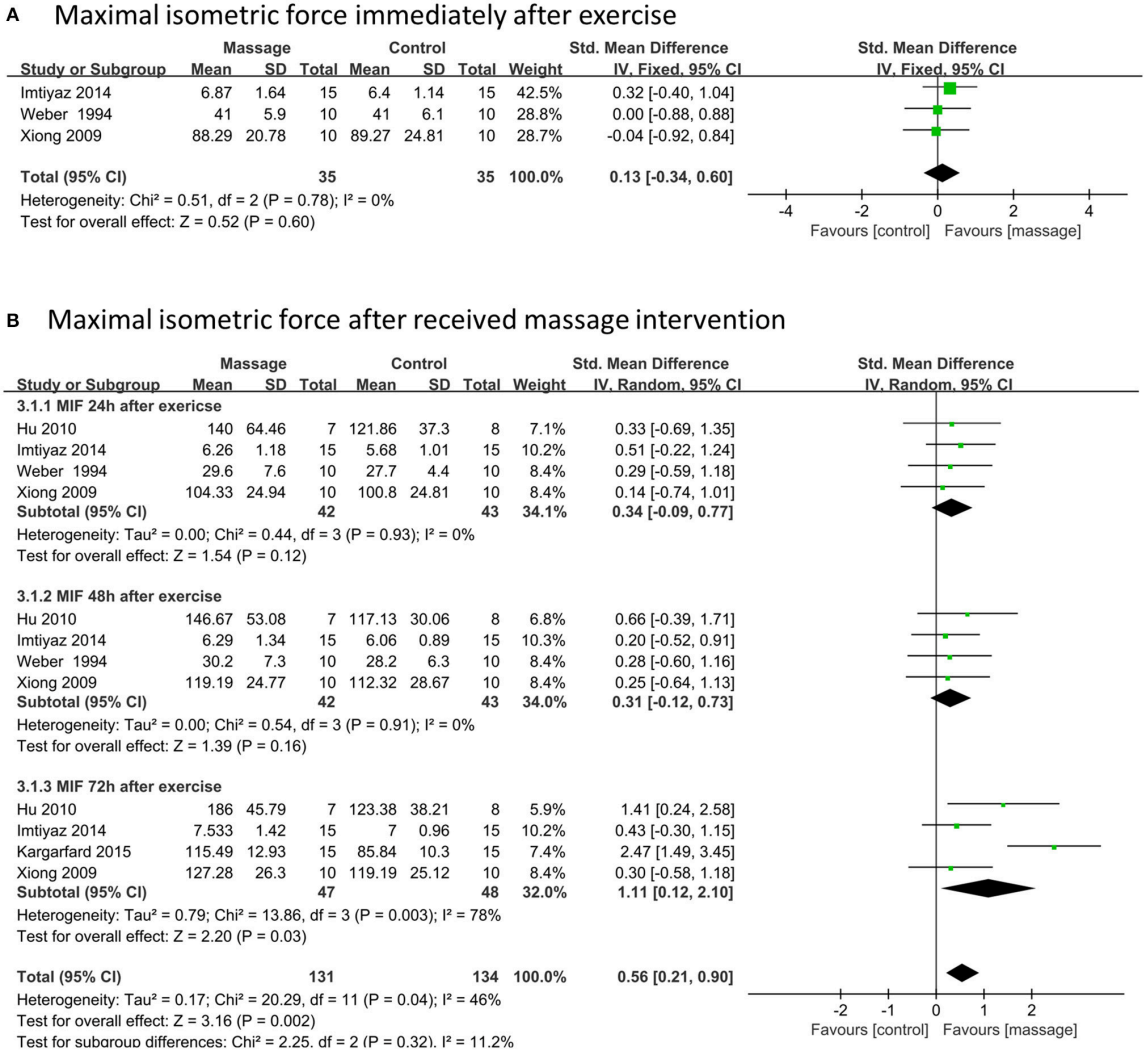
Meta-analysis of effects of massage intervention on MIF. **(A)** The time point immediately. **(B)** Time points after received massage intervention combined 24, 48, and 72 h after exercise. MIF, maximal isometric force.

Three data points with 70 participants compared MIF between massage and control groups immediately after intense exercise. No significant difference was observed based on the fixed model meta-analysis (SMD 0.13, 95%CI –0.34 to 0.60, *P* = 0.60, I^2^ = 0%) (Figure 4A). However, 16 data points (including 265 participants) demonstrated that massage intervention could significantly enhance the performance of MIF compared to controls (SMD 0.56, 95%CI 0.21–0.00, *P* = 0.002, I^2^ = 46%) (Figure 4B). Among these, only 72 h post-exercise subgroups showed significant positive effects of massage intervention on MIF (SMD 1.11, 95%CI 0.12–2.1, *P* = 0.03, I^2^ = 78%). No significant difference of MIF was observed between massage and control groups either at 24 h (SMD 0.34, 95%CI –0.09 to 0.77, *P* = 0.12, I^2^ = 0%) or 48 h (SMD 0.31, 95%CI –0.12 to 0.73, *P* = 0.16, I^2^ = 0%) post-exercise based on each subgroup meta-analysis.

Sensitivity analysis revealed that outcomes of the total effect of MIF after strenuous exercise were stable when removing trials one by one.

### Effects of massage on peak torque

**Figure 5:**
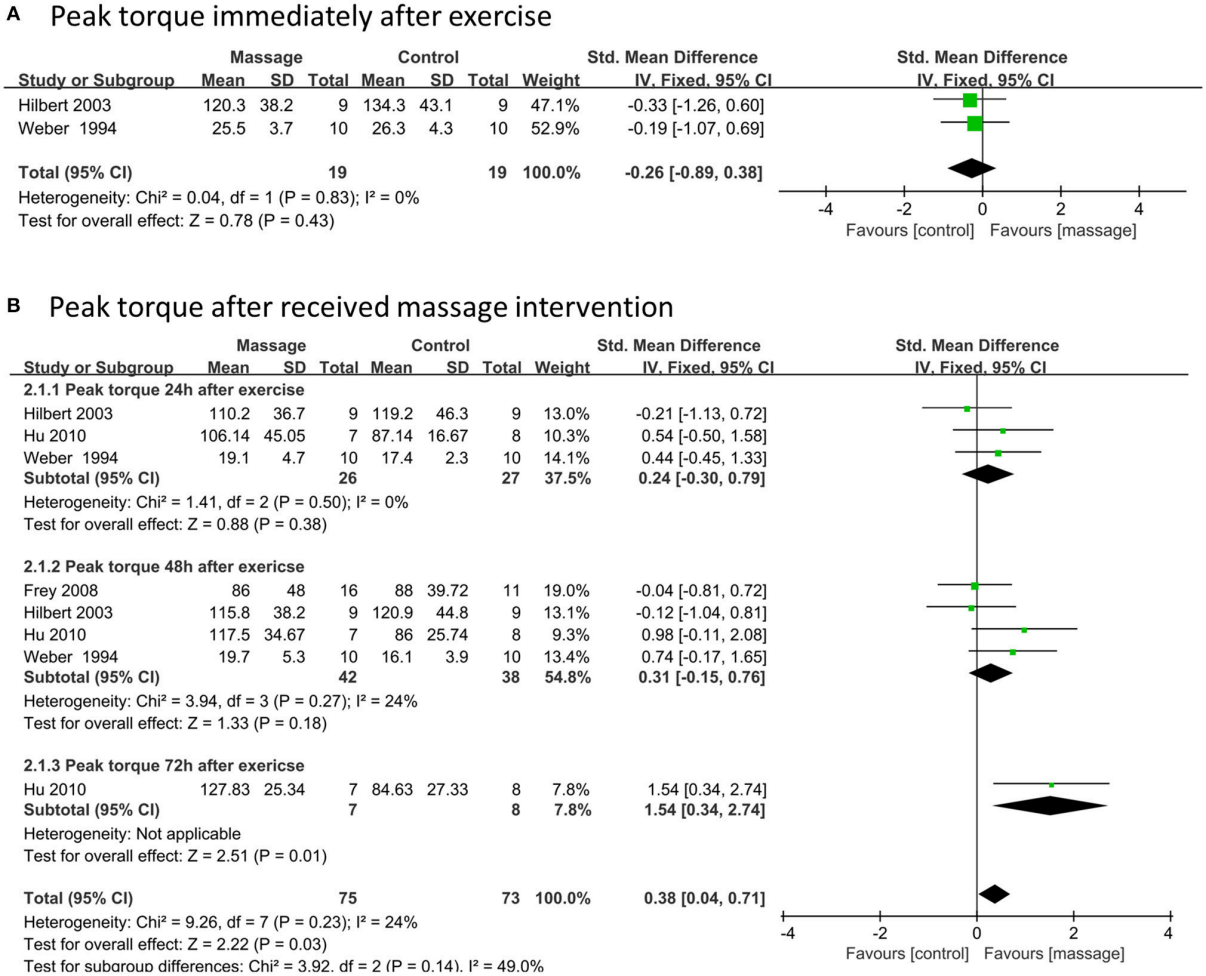
Meta-analysis of effects of massage intervention on peak torque. **(A)** The time point immediately. **(B)** Time points after received massage intervention combined 24, 48, and 72 h after exercise.

Two data points with 38 participants compared peak torque between massage and control groups immediately after exercise. No significant difference was observed based on the fixed model meta-analysis (SMD –0.26, 95%CI –0.89 to 0.38, *P* = 0.43, I^2^ = 0%) (Figure 5A). Eight data points (148 participants) as a total effect showed that massage intervention significantly increased peak torque after strenuous exercise when compared to the control group (SMD 0.38, 95%CI 0.04–0.71, *P* = 0.03, I^2^ = 24%) though no significant difference was found in the subgroups of 24 or 48 h (Figure 5B).

Sensitivity analysis demonstrated that the outcome of peak torque after strenuous exercise was reversed when one of the trials was removed (Hu et al., 2010).

### Effects of massage on serum CK level

**Figure 6:**
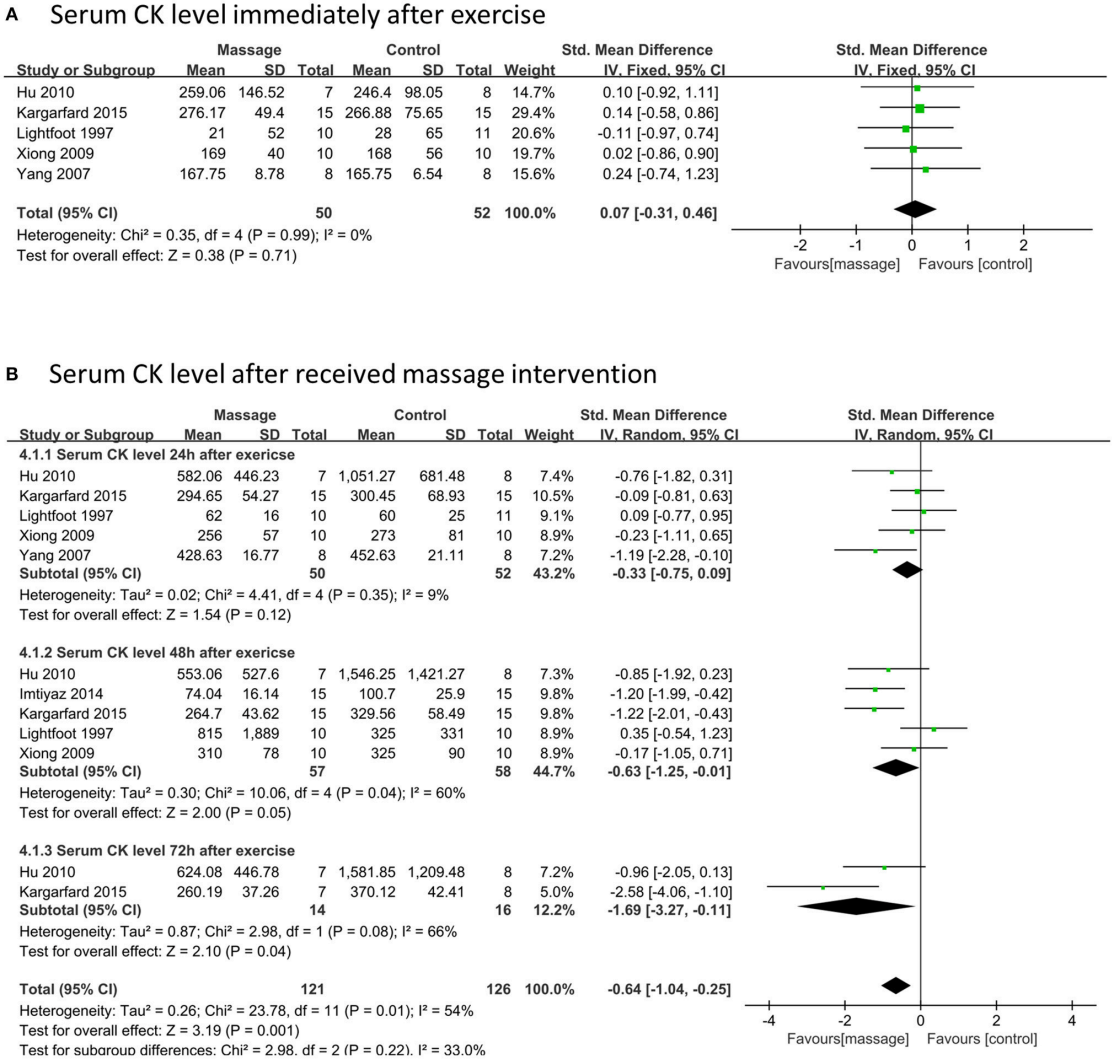
Meta-analysis of effects of massage intervention on the serum CK level. **(A)** The time point immediately. **(B)** Time points after received massage intervention combined 24, 48, and 72 h after exercise. CK, creatine kinase.

Five data points with 102 participants reported the outcome of serum CK level comparing massage and control groups immediately after exercise. No significant difference was found between the two groups (SMD 0.12, 95%CI –0.31 to 0.56, *P* = 0.58, I^2^ = 0%) based on fixed meta-analysis (Figure 6A). Seven articles including twelve data points (including 247 participants) showed that the serum CK level significantly decreased in the massage intervention group when compared to the control group (SMD –0.64, 95%CI –0.25 to –1.04, *P* = 0.001, I^2^ = 54%) as a total effect (Figure 6B). Among these, the subgroups of 48 and 72 h showed massage intervention could significantly decrease the serum CK level at 48 h (SMD –0.63, 95%CI –1.25 to –0.01, *P* = 0.05, I^2^ = 60%) and 72 h (SMD –1.69, 95%CI –3.27 to –0.11, *P* = 0.04, I^2^ = 66%) post-strenuous exercise. However, no significant difference was observed in the 24 h subgroup (SMD –0.33, 95%CI –0.75 to 0.09, *P* = 0.12, I^2^ = 9%).

Sensitivity analysis showed that outcomes of the total effects of the serum CK levels were stable when trials were removed one by one.

## Discussion

Massage therapy intervention is often used for reducing muscle pain or soreness (Field, 2016), and increasing post-exercise muscle performance (Buttagat et al., 2016; Best and Crawford, 2017). Several lines of evidence contributed to explaining the mechanisms of massage therapy on DOMS: (a) modulation of the activity of the parasympathetic nervous system (Weerapong et al., 2005), (b) increase in blood and lymphatic flow to rapidly clear the biochemical markers of muscle damage [e.g., CK and lactate dehydrogenase (LDH); Bakar et al., 2015], (c) psychophysiological response also plays an essential role in reducing pain (Arroyo-Morales et al., 2011).

Best et al. reviewed the effectiveness of massage on muscle recovery including 27 studies and 440 participants, showing little effect of massage on muscle recovery after intense exercise (Best et al., 2008). However, only six RCTs investigated DOMS and muscle function post-exercise, and in some of the studies, the data could not be extracted for meta-analysis since the published papers did not report the mean and SD. Thus, Best et al.'s review did not pool the extracted data in a meta-analysis. Torres et al. showed that massage could alleviate muscle soreness 24 h after intense exercise, but few trials were pooled in the meta-analysis (3 trials) (Torres et al., 2012). Other reviews also reported that self-myofascial release (a type of self-massage using a foam roller) could alleviate muscle pain and enhance muscle performance after strenuous exercise (Beardsley and Skarabot, 2015; Cheatham et al., 2015), but no trials were pooled into a meta-analysis to assess the effectiveness. Consistent with previous reviews, the present study confirmed that massage was an effective intervention for reducing DOMS after strenuous exercise with the outcomes of muscle soreness rating, muscle performance (MIF and Peak Torque) and the serum CK level. In addition, it is well known that the evidence from RCTs will be much better than from case studies. Therefore, this review pooled only RCTs in the meta-analysis with a total number of 23 data points (including 504 participants), to be better able to evaluate the effectiveness of massage on DOMS and muscle performance.

This systematic review and meta-analysis showed that the participants receiving massage intervention post-strenuous exercise experienced a reduction in muscle soreness rating as a total effect. Additionally, our findings demonstrated that the SMD of 48 and 72 h subgroups were –1.51 and –1.46, respectively. This result was greater than that of 24 h subgroups (–0.61), indicating that alleviating muscle pain from massage intervention would be the more efficacious at 48 and 72 h post-exercise compared to 24 h post-exercise. Moreover, the stability of the total effect sensitivity analysis also strengthened the results.

Previous systematic reviews demonstrated that there was little evidence supporting the use of massage to enhance muscle performance after strenuous exercise (Best et al., 2008; Torres et al., 2012). Poppendieck et al. observed that post-exercise manual massage increased recovery of sprint performance (Poppendieck et al., 2016). However, many articles included in this meta-analysis are not RCTs. in contrast to Best et al.'s and Torres et al.'s reviews, the current evidence showed that massage intervention increased MIF and peak torque after exercise as the total effects. These results may reflect more trials included that had positive effects of post-exercise massage on muscle performance. Three studies (Yang, 2007; Xiong et al., 2009; Hu et al., 2010) using Chinese traditional massage were pooled in this meta-analysis. The single effects of those trials were not different from those of the trials using Western massage. In addition, two studies (Xiong et al., 2009; Hu et al., 2010) used daily massage and one study (Mancinelli et al., 2006) applied massage 48 h post-exercise, however the effects of these single trials were not different from the others. Conversely, the effectiveness of single trials is not enough to show significant benefits of massage intervention on muscle performance post-strenuous exercise. Additional trials and participants are needed. Furthermore, contrary to Poppendieck et al.'s review, which included studies using endurance-type exercise, most of the studies included in this review used eccentric strength exercise to induce DOMS, which may also contribute to the difference between this meta-analysis and that of previous reviews.

The serum CK level was frequently considered a marker of inflammation and skeletal muscle damage influencing the recovery of muscle performance (Clarkson and Sayers, 1999; Romagnoli et al., 2015). Our findings showed that massage intervention decreased the serum CK levels as a total effect, suggesting that massage decreased inflammation and muscle damage and promoted muscle performance recovery. This result is consistent with the results of muscle soreness rating, MIF, and peak torque, supporting evidence of the positive physiological effects of massage therapy on DOMS. Fast clearance of serum CK level from the circulatory system was thought to be the reason that massage promotes muscle recovery and performance.

### Strength and limitations

This study is the first meta-analysis that combines both Chinese and Western massage at different time points after strenuous exercise, assessing whether massage intervention was effective in alleviating DOMS. Compared with previous studies, the present study included three articles using Chinese traditional massage intervention and more trials were pooled into the meta-analysis than in previous systematic reviews. This strategy implied greater evidence to evaluate the effectiveness of massage intervention after exercise. Furthermore, current evidence suggests that massage is not only effective in reducing muscle pain after intense exercise, but also in increasing muscle performance and reducing the serum CK level.

This review searched a wide variety of database including two Chinese electronic databases for relevant articles and included the trials performing Chinese traditional massage that have not previously been reviewed. Two authors independently searched and selected the included studies, extracted the data, and assessed the risk of bias of every trial using recommended protocols and methodological schemes. Therefore, the results of this meta-analysis are considered a significant contribution.

However, this meta-analysis has several limitations. First, the quality of all the included trials in this meta-analysis was low. None of the studies detailed allocation concealment although all the trials used a randomization method. None of the studies met the blinding of participants though it seems unfeasible to use the blinding method in view of the massage intervention. This factor increased the selection bias and performance bias. Only two articles (18.2%) masked their outcome assessors, which increased the risk of detection bias. Second, the trials varied in methodological design (i.e., exercise type, control intervention), massage type, and conditions (i.e., duration). Thus, the outcomes of muscle soreness have high heterogeneity although the total effects of outcomes were stable. Sensitivity analysis implied that the outcome of peak torque after strenuous exercise was unstable; therefore, the results should be considered with caution. Third, there may be some publication bias as unpublished articles could not be searched in this review although the funnel plot asymmetry did not show the bias. Finally, the number of trials and participants was relatively small; therefore, larger sample sizes in future studies are needed to better understand the effects of massage intervention on DOMS and muscle performance.

## Conclusion

This systematic review and meta-analysis demonstrated that massage intervention could be effective for alleviating DOMS, as well as increasing muscle performance after strenuous exercise. The highest efficacy was achieved at 48 h post-exercise. Massage is a useful and practical therapy for exercise participants or athletes. Nevertheless, it is necessary to be cautious about the results in view of the limitations outlined in the present study. More RCTs with large sample sizes are needed for better understanding the effectiveness of massage intervention on DOMS and muscle performance.

## Author contributions

XC and JZ designed the systematic review and supervised the entire program; JG and LL reviewed all the studies and extracted the information from the eligible trials; YG analyzed the data and prepared the figures and table; XC, JG, and LL wrote the paper; XC, RZ, and JX revised the manuscript. All authors reviewed and approved the manuscript.

### Conflict of interest statement

The authors declare that the research was conducted in the absence of any commercial or financial relationships that could be construed as a potential conflict of interest.
