# Supplementary material for: Massage Alleviates Delayed Onset Muscle Soreness after Strenuous Exercise: A Systematic Review and Meta-Analysis
Source: Front Physiol. 2017 Sep 27;8:747. doi: 10.3389/fphys.2017.00747 (PMC5623674; doi:10.3389/fphys.2017.00747)
Supplement: Supplementary file 1 [file DataSheet1.DOCX]

Massage Alleviates Delayed Onset Muscle Soreness After Strenuous Exercise: A Systematic Review and Meta-Analysis

Jianmin Guo^1,2,#^, Linjin Li^3,#^ , Yuxiang Gong^1^, Jun Zou^1*^ and Xi Chen^2,4,*^.

***Correspondence:** Corresponding Author:

E-mail: junzou@sus.edu.cn

E-mail: chenab004@126.com

**1 Supplementary Data**

Supplementary 1 (S1). The details of search strategies in this study

**2 Supplementary Figures**

Supplementary Figure 1(SFig 1). The Funnel plot of muscle soreness rating.

Supplementary Figure 2(SFig 2). The Funnel plot of maximal isometric force(MIF).

Supplementary Figure 3(SFig 3). The Funnel plot of the serum CK level.
