# Supplementary material for: Massage Alleviates Delayed Onset Muscle Soreness after Strenuous Exercise: A Systematic Review and Meta-Analysis
Source: Front Physiol. 2017 Sep 27;8:747. doi: 10.3389/fphys.2017.00747 (PMC5623674; doi:10.3389/fphys.2017.00747)
Supplement: Supplementary file 2 [file DataSheet2.DOC]

Massage Alleviates Delayed Onset Muscle Soreness After Strenuous Exercise: A Systematic Review and Meta-Analysis

**Search strategies for all databases**

1. Search Strategy for pubmed:

#1 ((((((exercise) OR muscle， skeletal) OR athletic injuries) OR soft tissue injuries) OR Creatine kinase) OR muscle fatigue) OR muscle weakness (554425)

#2 DOMS (765)

#3 ((damage) OR injury) OR injuries (1549294)

#4 muscle (941534)

#5 #3 and #4 (103101)

#6 muscle soreness (7041)

#7 #1 or #2 or #5 or #6 (628084)

#8 massage (12429)

#9 randomized OR random OR randomly OR randomized controlled trail OR controlled clinical trail (983227)

#10 (animal) NOT huaman (5857628)

#11 #9 NOT #10 (827230)

#12 #7 and #8 and #11 (296)

2. Search Strategy for Cochrane library:

#1 injuries OR injury OR creatine kinase OR soreness OR damage (41153)

#2 fatigue or weakness (18185)

#3 #1 or #2 (57335)

#4 exercise AND muscle (10555)

#5 #3 AND #4 (3246)

#6 random or randomized or randomization or control or controlled (959477)

#7 massage (2602)

#8 #5 AND #6 AND #7 (140)

3. Search Strategy for EMbase:

#1 exercise or sports (535084)

#2 injuries OR injury OR creatine kinase OR soreness OR damage or fatigue or weakness (890549)

#3 muscle (1285349)

#4 #1 AND #2 AND #3 (21304)

#5 random OR randomized OR randomization OR control OR controlled (7475795)

#6 massage (18826)

#7 #4 and #5 and #6 (92)

4. Search Strategy for EBSCO：

S1 exercise or sports (5254401)

S2 injuries OR injury OR creatine kinase OR soreness OR damage or fatigue or weakness (3476397)

S3 muscle (989730)

S4 S1 AND S2 AND S3 (34837)

S5 random OR randomized OR randomization OR control OR controlled (8234771)

S6 massage (43470)

S7 S4 and S5 and S6 (90)

5. Search Strategy for Web of science:

#1 exercise or sports (596878)

#2 injuries OR injury OR creatine kinase OR soreness OR damage or fatigue or weakness (2407615)

#3 muscle (1699989)

#4 #1 AND #2 AND #3 (32396)

#5 random OR randomized OR randomization OR control OR controlled (7624815)

#6 massage (15952)

#7 #4 and #5 and #6 (85)

6. Search Strategy for CNKI (19)

FT=随机+对照+随机对照+随机对照研究+随机对照试验+随机分配 AND SU=推拿+按摩+放松 AND SU=延迟性肌肉酸痛+延迟性肌肉疼痛+运动后肌肉酸痛+运动后肌肉疼痛+DOMS+运动后肌肉损伤+延迟性肌肉损伤

7. Search Strategy for wanfang database (100)

主题：（随机+对照+随机对照+随机对照研究+随机对照试验+随机分配） AND 主题：（推拿+按摩+放松） AND 主题：（延迟性肌肉酸痛+延迟性肌肉疼痛+运动后肌肉酸痛+运动后肌肉疼痛+DOMS+运动后肌肉损伤+延迟性肌肉损伤）
