# Supplementary figures and images for: Massage Alleviates Delayed Onset Muscle Soreness after Strenuous Exercise: A Systematic Review and Meta-Analysis
Source: Front Physiol. 2017 Sep 27;8:747. doi: 10.3389/fphys.2017.00747 (PMC5623674; doi:10.3389/fphys.2017.00747)

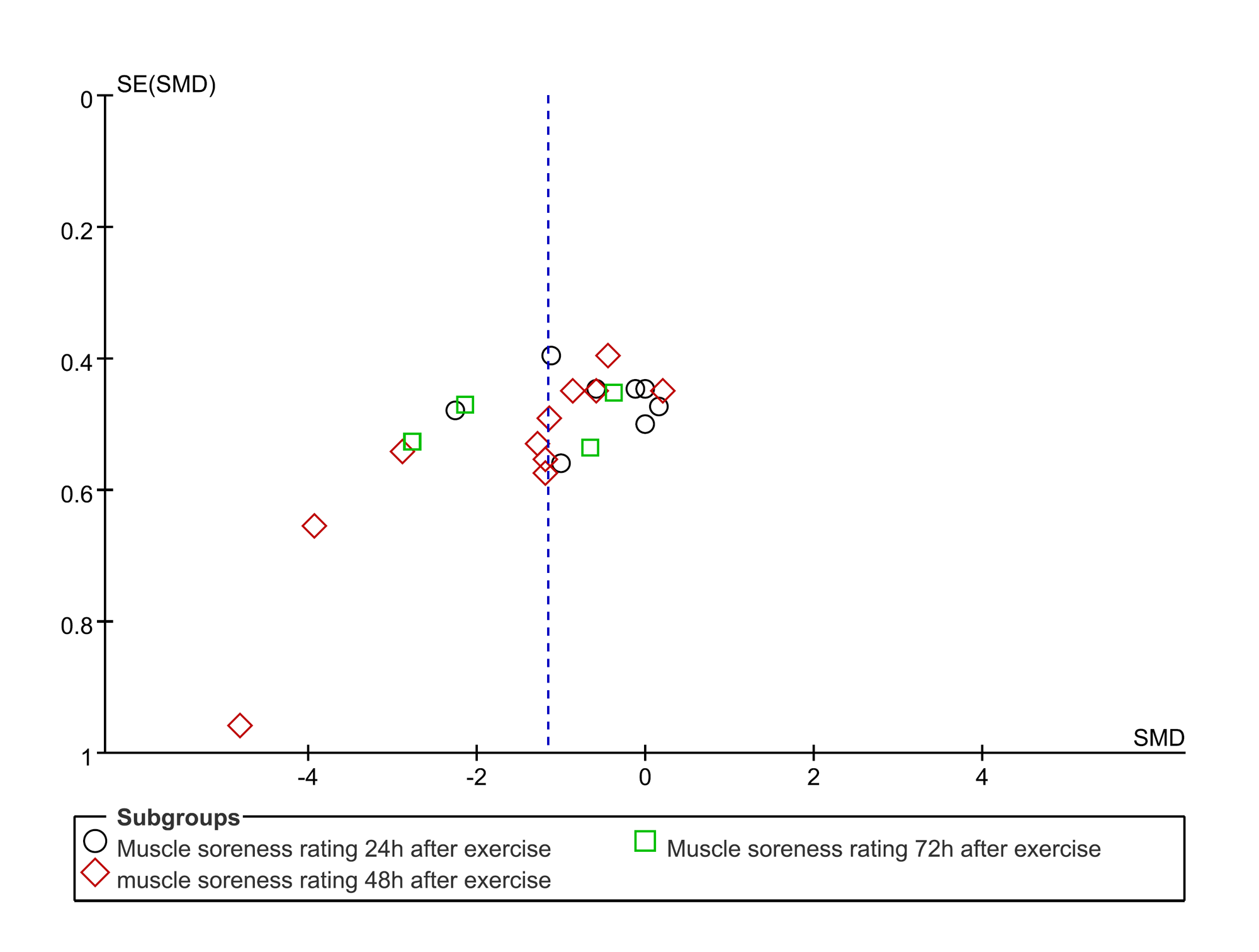

Supplement: Supplementary file 3 [file Image1.TIF]

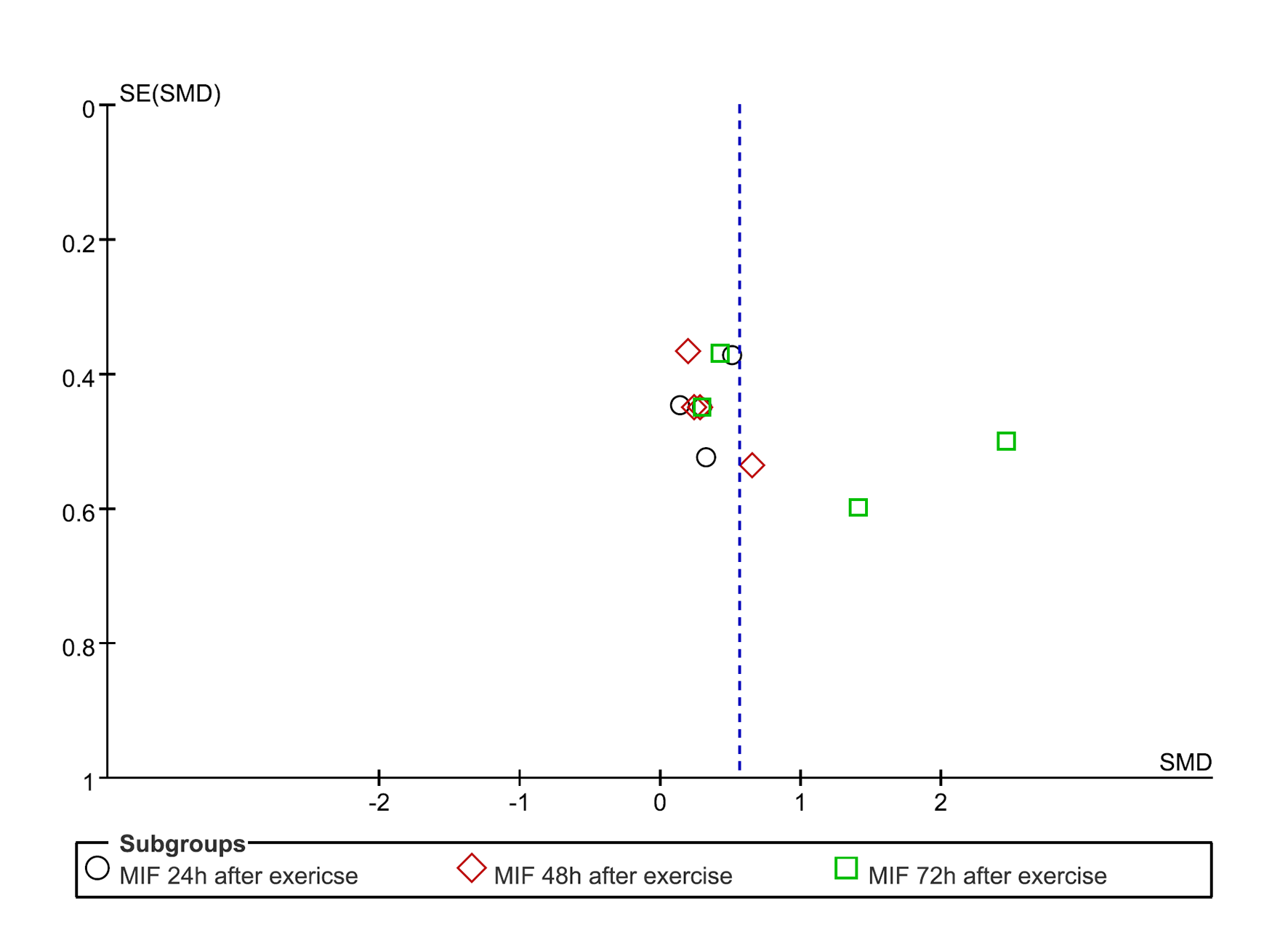

Supplement: Supplementary file 4 [file Image2.TIF]

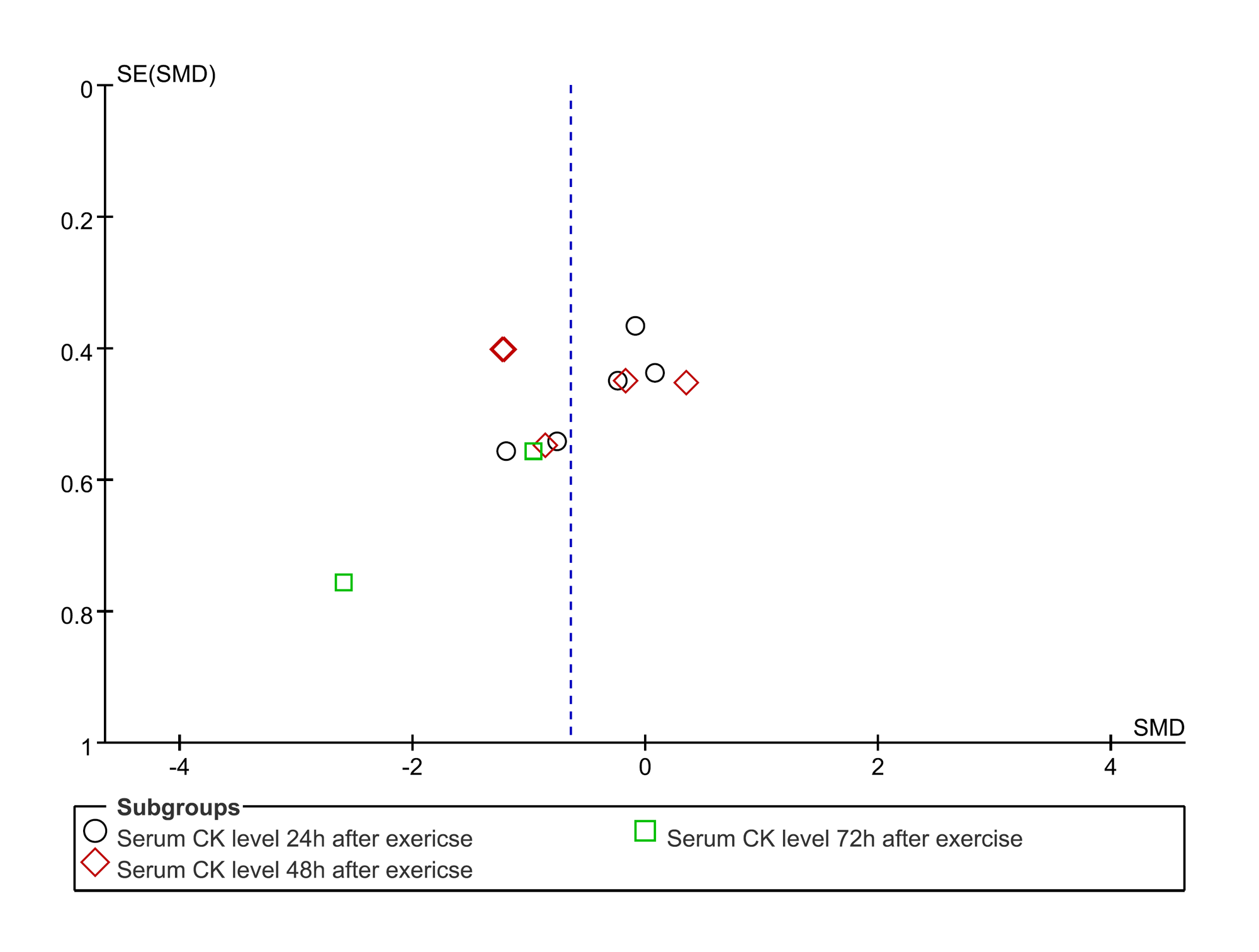

Supplement: Supplementary file 5 [file Image3.TIF]
